# Genome-wide epistasis and co-selection study using mutual information

**DOI:** 10.1093/nar/gkz656

**Published:** 2019-07-30

**Authors:** Johan Pensar, Santeri Puranen, Brian Arnold, Neil MacAlasdair, Juri Kuronen, Gerry Tonkin-Hill, Maiju Pesonen, Yingying Xu, Aleksi Sipola, Leonor Sánchez-Busó, John A Lees, Claire Chewapreecha, Stephen D Bentley, Simon R Harris, Julian Parkhill, Nicholas J Croucher, Jukka Corander

**Affiliations:** 1 Department of Mathematics and Statistics, Helsinki Institute for Information Technology (HIIT), Faculty of Science, University of Helsinki, FI-00014 Helsinki, Finland; 2 Department of Computer Science, Aalto University, Espoo, FI-00014, Finland; 3 Division of Informatics, Faculty of Arts and Sciences, Harvard University, Cambridge, MA 02138, USA; 4 Parasites and Microbes, Wellcome Sanger Institute, Cambridge, CB10 1SA, UK; 5 Department of Biostatistics, University of Oslo, Oslo, 0317, Norway; 6 Department of Microbiology, New York University School of Medicine, New York, NY 10016, USA; 7 Department of Medicine, University of Cambridge, Cambridge CB2 0QQ, UK; 8 Bioinformatics & Systems Biology program, King Mongkut's University of Technology Thonburi, Bangkok 10150, Thailand; 9 Department of Veterinary Medicine, University of Cambridge, Madingley Road, Cambridge, CB3 0ES, UK; 10 MRC Centre for Global Infectious Disease Analysis, Department of Infectious Disease Epidemiology, St. Mary's Campus, Imperial College London, London, W2 1PG, UK

## Abstract

Covariance-based discovery of polymorphisms under co-selective pressure or epistasis has received considerable recent attention in population genomics. Both statistical modeling of the population level covariation of alleles across the chromosome and model-free testing of dependencies between pairs of polymorphisms have been shown to successfully uncover patterns of selection in bacterial populations. Here we introduce a model-free method, SpydrPick, whose computational efficiency enables analysis at the scale of pan-genomes of many bacteria. SpydrPick incorporates an efficient correction for population structure, which adjusts for the phylogenetic signal in the data without requiring an explicit phylogenetic tree. We also introduce a new type of visualization of the results similar to the Manhattan plots used in genome-wide association studies, which enables rapid exploration of the identified signals of co-evolution. Simulations demonstrate the usefulness of our method and give some insight to when this type of analysis is most likely to be successful. Application of the method to large population genomic datasets of two major human pathogens, *Streptococcus pneumoniae* and *Neisseria meningitidis*, revealed both previously identified and novel putative targets of co-selection related to virulence and antibiotic resistance, highlighting the potential of this approach to drive molecular discoveries, even in the absence of phenotypic data.

## INTRODUCTION

Comparative methods for detecting co-evolutionary signals from population sequence data have received a lot of attention over the last few decades. As one of the more striking examples, statistical analysis of covariation between non-adjacent sites in large protein alignments has proven effective for predicting contacts between sites in the three-dimensional protein structure (1–8). Since sites in contact in the protein structure co-evolve under a common structural constraint, they give rise to a detectable trace of correlation in the protein alignment. Similarly, sites co-evolving under a shared selective pressure may give rise to a co-selection pattern that can be detected from sequence alignments, even in the absence of appropriate phenotypic data. As a result, attention has recently been directed toward exploratory co-variation analysis of genome-wide nucleotide alignments for bacterial populations, where the aim is to reveal putative sites co-evolving under a shared selective pressure and possibly, but not necessarily, being involved in epistatic interactions (9–11).

Genome-scale analysis of co-variation at single-nucleotide resolution, here termed as genome-wide epistasis and co-selection study (GWES), has already shown great potential; however, it poses considerable statistical and computational challenges as the number of pairs to be considered increases quadratically with the number of sites. Previous GWES approaches have been based on either straightforward pairwise tests (9), which do not distinguish between indirect and direct interactions, or a more elaborate model-based technique known as direct coupling analysis (DCA) (10,11), which is computationally demanding. The main motivation behind pairwise structure learning methods has typically been scalability; however, a recent simulation study with synthetic network models showed that pairwise methods based on mutual information (MI) can be as accurate as and even outperform model-based methods in the high-dimensional regime (12), which is the typical setting in GWES. While MI has been successful in detecting co-evolution from protein and RNA data (1,2,13,14), it has not yet been systematically applied to bacterial population genomics at a genome-wide scale.

In this work we introduce a novel MI-based GWES method, SpydrPick, which is scalable to handle analyses even at a pan-genome-wide scale. To account for population structure, we use a sequence reweighting technique commonly employed when analyzing protein sequence alignments (4,5,14), and also more recently when performing GWES (10,11). To select the best candidates of directly co-selected or interacting mutations among the identified signals of co-variation, we use a pruning method originally introduced for analysis of gene expression data (15), combined with an outlier detection method that identifies significant outliers in terms of a global background distribution estimated across the genome. The focus on the statistical quantification of the background pattern across the genome lends itself well to an intuitive and efficient visualization of the results akin to a Manhattan plot used in genome-wide association studies, which we term as the GWES Manhattan plot. We demonstrate the usefulness and reliability of SpydrPick by application to both simulated data and two large population genomic datasets of the major human pathogens *Streptococcus pneumoniae* and *Neisseria meningitidis*. For the latter pathogen, we analyzed the entire pan-genome, which contains so many mutations that most model-based approaches are computationally infeasible, including even our recent highly optimized DCA-based software (11). An open-source C++ implementation of SpydrPick is available at https://github.com/santeripuranen/SpydrPick.

## MATERIALS AND METHODS

### Method

**Figure 1.**
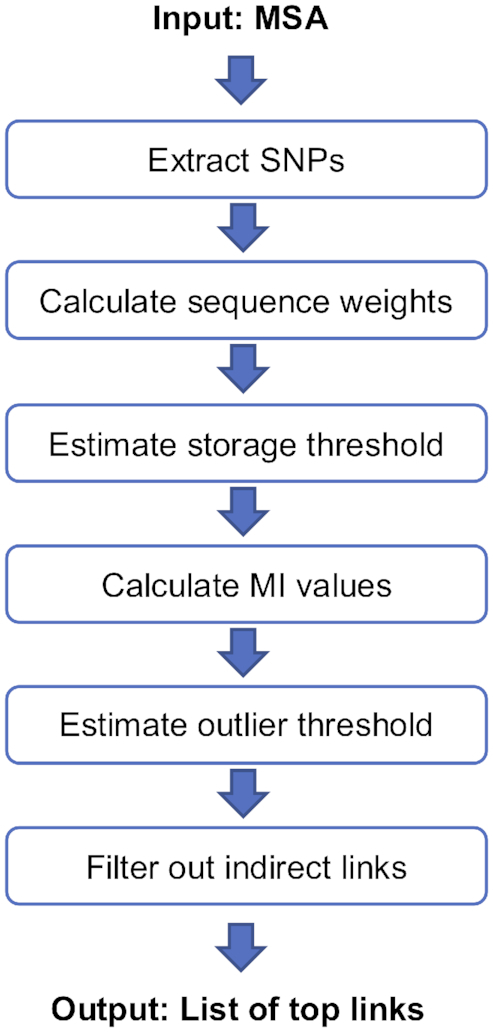
An overview of the SpydrPick pipeline.

An overview of the SpydrPick pipeline is shown in Figure 1. The different steps are described in detail in the following sections.

#### Mutual information

MI is an information theoretic measure of the mutual dependence between two random variables (16). More specifically, let $X$ and $Y$ be two discrete random variables with outcome spaces $val( X )$ and $val( Y )$. The MI between $X$ and $Y$ is then formally defined by:

(1) $$\begin{equation*}MI\left( {X,Y} \right) = \mathop \sum \limits_{x \in val\left( X \right)} \mathop \sum \limits_{y \in val\left( Y \right)} p\left( {x,y} \right)\log \left( {\frac{{p\left( {x,y} \right)}}{{p\left( x \right)p\left( y \right)}}} \right),\end{equation*}$$

where $p( {x,y} )$ is the joint probability of $X\ = \ x$ and $Y\ = \ y$, while $p( x ) = \mathop \sum \limits_{y \in val( Y )} p( {x,y} )$ and $p( y ) = \mathop \sum \limits_{x \in val( X )} p( {x,y} )$ are the corresponding marginal probabilities. In practice, the distributions in (1) are typically unknown and have to be estimated from data. Let $n( {x,y} )$ denote the count of the joint outcome $X\ = \ x$ and $Y\ = \ y$ occurring in a dataset containing $n$ independent and identically distributed (IID) observations generated from $p( {X,Y} )$. Typically, the joint probabilities are estimated by the relative frequencies of the joint outcomes corresponding to maximum likelihood estimates. To avoid issues related to zero counts and increase the stability of the estimator, we add 0.5 to the joint counts:

(2) $$\begin{equation*}\hat{p} \left( {x,y} \right) = \frac{{n\left( {x,y} \right) + 0.5}}{{n + {r_X}{r_Y} \cdot 0.5}},\end{equation*}$$

where ${r_X} = | {val( X )} |$ and ${r_Y} = | {val( Y )} |$. The corresponding marginal probabilities are calculated from the estimated joint probabilities as described above. In the Bayesian framework, the above point estimator is the posterior mean under a Dirichlet prior distribution with the hyperparameters set to 0.5, corresponding to Jeffreys’ prior (17).

#### Sequence reweighting

In the context of this work, $X$ and $Y$ in the previous paragraph correspond to single-nucleotide polymorphisms (SNPs) and the outcome spaces represent (subsets of) the four nucleotides $A,\ C,\ G,\ T$ and an additional category representing gaps. The observed data is in form of a multiple sequence alignment (MSA) containing $n$ sequences $( {{S_1}, \ldots ,{S_n}} )$ of length $L$. In general, the sequences in an MSA strongly violate the IID assumption since they share a linkage through an evolutionary relationship. This non-independence has long been recognized as a major issue in comparative analysis, introducing a phylogenetic bias that leads to an increase in false positives (18), impeding the separation of interesting signals from background noise caused by the population structure. As a result, various techniques for correcting for the population structure have been developed over the years (see (13) for an overview). Here, we apply a technique known as sequence reweighting, which has successfully been used previously for both protein contact map prediction (4,5) and DCA-based GWES (10,11). Reweighting assigns a weight to each sequence according to how different it is from the other sequences in the MSA, such that the counts of allele pairs occurring in the MI estimator will reflect the level of clusteredness across the MSA.

Let ${m_i}$ denote the number of sequences (including ${S_i}$) in the data whose mean per-site Hamming distance to ${S_i}$ is smaller than a specified threshold. The weight $w_i$ given to sequence ${S_i}$ is then simply calculated by

$$\begin{equation*}w_i = \frac{1}{m_i}.\end{equation*}$$

Similar to previous works (5,10,11), we use a default distance threshold value of 0.1. Considering the large genetic distance separation that was recently observed for many bacterial species (19), we expect the results to be fairly robust toward the exact value of the distance threshold, as long as the value is chosen from an appropriate region. For example, previous DCA-based methods have been shown to be stable for values in the range of 0.10–0.25 (5,10).

The effective count ${n_{{\rm{eff}}}}( {x,y} )$ is calculated by summing the weights of all sequences with the corresponding joint configuration over the SNP sites represented by $X$ and $Y$. The counts in (2) are then replaced with the corresponding effective counts:

$$\begin{equation*}\hat{p}_{\rm{eff}} \left( {x,y} \right) = \frac{{n_{\rm{eff}}\left( {x,y} \right) + 0.5}}{{n_{\rm{eff}} + {r_X}{r_Y} \cdot 0.5}}.\end{equation*}$$

The above estimates are finally plugged into (1) resulting in the reweighted MI estimator.

#### Filtering out indirect links

An unavoidable issue with methods based solely on pairwise association tests is their inability to distinguish between direct and indirect associations. In particular, in the GWES context it is typically expected that a strong direct dependence between two distant SNP sites would be accompanied by a collection of slightly weaker indirect dependencies between sites in close proximity of the coupled sites due to genetic linkage. As a result, pinpointing the exact locations of co-evolving loci at SNP resolution in a bacterial GWES is in general very difficult due to strong LD between nearby sites. Still, considering that the identified links need to be examined manually, our aim is to produce as compact a list of SNP pairs as possible, containing the most likely candidates of mutations co-evolving under a shared selective pressure.

To select a subset of SNP pairs containing only the most promising links, we use the same filtering technique as ARACNE, which was originally introduced as a method for inferring gene expression networks (15). The filtering technique is based on a property known as the data processing inequality, which states that if two variables $X$ and $Y$ only interact through a third variable $Z$, then

$$\begin{equation*} MI(X,Y) \leq \min\left[MI(X,Z),MI(Z,Y) \right].\end{equation*}$$

**Figure 2.**
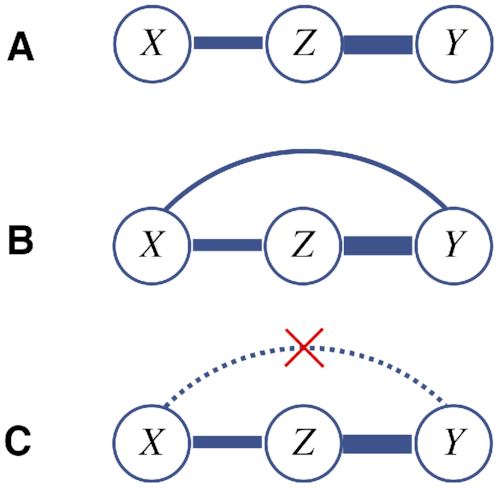
Illustration of the ARACNE step (the width of the links represents the interaction strength): (**A**) true interaction structure: *Z* is strongly linked to *X* and *Y*, which are not directly linked to each other. (**B**) A pairwise test outputs a significant association between *X* and *Y* due to the indirect link through *Z*. (**C**) The ARACNE step removes the indirect link between *X* and *Y*, being the weakest out of the three links.

In other words, the indirect dependence between $X$ and $Y$ cannot be larger than either of the two direct dependencies through which it is mediated. Formally, ARACNE starts from a graph containing a link for each non-zero MI value. The algorithm then examines each triplet of mutually linked variables and removes the weakest link (see Figure 2). In the degenerate case, where there is no unique weakest link in a triplet, no link is removed. The algorithm is order-independent in the sense that a link that has been marked for removal from one triplet is still considered present with respect to a non-examined triplet containing that link.

Naively applying the ARACNE filtering step would be computationally intractable, since there are in total $\binom{L}{3}$ possible triplets. However, in practice it is sufficient to run the procedure over a small list containing only the top estimated links. Consequently, the main computational part will still be to estimate the MI values over the $\binom{L}{2}$ pairs. The ARACNE approach is not only appealing due to its computational simplicity, but also its ability to produce a small representative set of links that are most likely to be direct. One of the drawbacks with this approach is that it will never output a triplet of mutually linked sites (except in the degenerate case) even if such a triplet existed. However, three mutually linked sites will still be contained in a single connected component and thus the association between the sites will remain visible.

#### Threshold for result storage

Saving the complete output of a GWES to disk would typically result in such large files that they would become unwieldy. Nevertheless, since the main target is to identify the largest MI values, estimation results can be filtered online (i.e. as each new value is calculated) to reduce the amount of storage required. To this end, we use a subsampling procedure to determine a threshold for saving a user-specified top fraction of the MI values. This is done by randomly selecting a subset of SNP pairs for which the MI values are calculated. The empirical cumulative distribution function is then used to estimate an appropriate saving threshold that corresponds to the user-specified top fraction. To increase stability, the procedure is repeated several times and the median threshold value is selected.

#### Outlier analysis

To assess if a link is strong enough to warrant further study, we perform an outlier analysis. Due to genetic linkage, SNPs in close chromosomal proximity tend to be in strong linkage disequilibrium (LD). Note that LD here refers to SNPs showing a significant association specifically due to close genetic linkage. Since strong LD masks any potential signal of shared co-evolutionary selection pressure, we restrict the outlier analysis to non-LD pairs. The default approach for filtering out LD-pairs is to use a simple distance-based cut-off.

To estimate an outlier threshold among the non-LD pairs, we use a data-driven procedure based on Tukey's outlier test (20). The test assesses how extreme an MI value is in comparison to a global background distribution observed for the analyzed dataset. If the MI value of a direct link is flagged as an outlier, the corresponding SNP pair will automatically be carried forward for further analysis. As background distribution for the outlier test, we use an extreme value distribution by which we effectively attempt to model the distribution of maximum MI values for a site (w.r.t. non-LD pairs). In practice, we save the maximum MI value of each site and calculate the lower (${Q_1}$) and upper (${Q_3}$) quartiles of the empirical extreme value distribution. Following Tukey's criterion, we then label an MI value larger than ${Q_3} + 1.5\ \times \ ( {{Q_3} - {Q_1}} )$ as an outlier. In addition to the default threshold, we label an MI value larger than ${Q_3} + 3\ \times \ ( {{Q_3} - {Q_1}} )$ as an extreme outlier.

The typical approach for determining significance in this type of problem is to run a permutation analysis (15,21). For this application, such an approach would be too inclusive since the maximum MI values observed in the background distribution of real MSAs exceed those observed under a null model in which the sites are unlinked through permutations. Moreover, the extent of the tail region of the background distribution may vary significantly between datasets due to differences in population structure, recombination rate, etc. For these reasons, our significance analysis is based on identification of outliers among the actual MI values observed for a particular population. Being based on quartiles, Tukey's outlier test is by design very robust against extreme values. The critical assumption behind this procedure is that the majority of SNPs are not linked to other SNPs beyond LD, which is a reasonable assumption in most cases.

#### Mutual information without gaps

When calculating the MI values, gaps are by default considered an outcome. While some gaps can be informative, others may simply be due to difficulties in the sequencing process: difficult-to-sequence regions may be systematically absent from all lower-quality sequences, resulting in distinct patches of gap characters that appear in parallel across samples. Hence, some interactions may be artificially amplified in regions with low-quality sequence data. To facilitate discovery of such cases in the subsequent manual analysis, we also calculate the MI value of the top pairs using only sequences where neither site of a pair contains a gap. Since the collection of sequences without gaps varies between pairs, it is difficult to compare gap-free MI values between SNP pairs in a meaningful way. However, the gap-free MI value can still be informative for a given pair in the sense that a large decrease in MI when dropping the gap sequences is an indication of a gap-driven interaction.

#### Implementation

The complete SpydrPick pipeline was implemented in C++ and supports parallel execution in a shared memory environment. Its space-efficient data structure, indexing strategy and online filtering of output jointly enable excellent scalability to an order of magnitude larger genome datasets than previous GWES software.

#### GWES Manhattan plot

For compactly visualizing the results of a GWES, we use a modified version of the GWAS Manhattan scatter plot. In a standard GWAS Manhattan plot, the association strength between a SNP and some phenotype (y-axis) is plotted against the chromosomal location of the SNP (x-axis), meaning that each point represents a single SNP. A GWES Manhattan plot has a similar design, however, each point now represents a pair of SNPs such that the x-axis displays the distance between the chromosomal locations of the SNPs and the y-axis displays the association strength between the SNPs, which is here determined by their MI value.

### Data

#### Simulated data

To test the accuracy of our method in a controlled setting, we applied it to simulated evolutionary scenarios with known parameter values. These simulated datasets were generated using fwdpp (22) along with custom functions to simulate bacterial recombination and selection.

To model a single recombining species adapting to multiple niches, we simulated two subpopulations, or demes, of size ${N_1}$ and ${N_2}$ that experience divergent selection pressures but exchange DNA for homologous recombination. We simulated a 200 kbp segment from a metapopulation of size ${N_1} + \ {N_2} = \ 100 000$ individuals, with an overall population mutation rate of $\theta \ = \ 2( {{N_1} + {N_2}} )\mu \ = \ 0.02$ per bp, where $\mu$ is the physical mutation rate. Each new mutation had a $5\ \times {10^{ - 6}}$ chance of affecting fitness and experiencing either positive selection in deme 1, or negative selection in deme 2. Fitness effects were multiplicative, with individual fitness calculated as $w\ = {( {1 + ds} )^m}$, where $s$ represents the selection coefficient of the $m$ mutations that have a fitness effect, and $d$ takes on a value of 1 or −1 depending on whether the individual is in deme 1 or 2, respectively.

Since our ability to detect the positively co-selected mutations in deme 1 may vary with its relative size in the metapopulation, we varied the size of deme 1. We explored scenarios in which demes were similar in size (50:50) or where deme 1 was considerably more rare, only 10 and 5% of the total metapopulation (10:90 and 5:95, respectively). For each parameter set, we specified a selection coefficient $s$ so that ${N_1}s\ = \ 100$.

We also varied the population recombination rate $\rho \ = \ 2( {{N_1} + {N_2}} )r$, where $r$ represents the physical recombination rate, so that mutations with fitness effects were either more linked ($\rho /\theta = \ 0.5,\ 1$) or less linked ($\rho /\theta = \ 2,\ 4$) to neutral mutations. Each individual in the metapopulation had the same chance of receiving DNA. Recombination within and between demes was proportional to deme size, such that the probability an individual in deme 1 served as a donor for any given recombination event was ${N_1}/( {{N_1} + {N_2}} )$, representing a scenario in which there are no significant physical barriers between the demes. For all simulations, individuals that served as recombination donors transferred geometrically distributed DNA tracts with a mean length of 500 bp.

For each scenario, we ran five simulations for $5( {{N_1} + {N_2}} )$ generations, after which a random sample of size $0.1( {{N_1} + {N_2}} ) = \ 10 000$ was taken from the metapopulation, sampling demes with respect to their relative sizes. To investigate how the sample size affected the accuracy of our method, we subsampled the initial dataset with sample sizes ranging from 50 to 800. Using 10 iterations per sample size, we thus generated $5\ \times \ 10\ = \ 50$ datasets for each scenario and sample size. In total, the simulation study covered 3000 datasets, which on average contained 14 976 SNPs, after filtering out sites with a minor allele frequency (MAF) <1%. On average, 10 mutations were randomly placed under selection in a simulation.

#### Streptococcus pneumoniae

Our first real alignment contained 3042 *S. pneumoniae* strains collected in Maela, a refugee camp close to the border between Thailand and Myanmar (23). The whole genome alignment was generated from short-read data aligned to the reference sequence of *S. pneumoniae* ATCC 700669 whose genome is a circular chromosome of 2 221 315 bp (24). Loci with MAF >1% and gap frequency (GF) <15% were included in the analysis. The filtered alignment contained 94 880 SNPs.

The diverse population structure in the data, together with the recombinant nature of *S. pneumoniae*, make the data ideal for GWES (10). Moreover, this particular dataset has previously been analyzed by DCA approaches, which successfully discovered several interacting regions with plausible biological explanations (10,11). Hence, the main aim for this dataset was to investigate how well the earlier highlight findings could be rediscovered using our model-free method.

#### Neisseria meningitidis

Our second real alignment contained 2148 *N. meningitidis* strains, of which 543 were published by Lucidarme *et al.* (25) and the rest were obtained from different sequencing projects run in the Wellcome Sanger Institute, Cambridge (Supplementary Table S1). The pan-genome of the strains included in the study was created using Roary (26), with a percentage of isolates needed to consider a gene as core set to 95%. The core gene alignment and individual gene alignments of the 13 052 genes conforming the pan-genome under the above criteria were obtained directly from the output. All individual genes were concatenated to obtain a pan-genome-wide alignment of 11 375 926 bp using the Alignment Manipulation and Summary (AMAS) tool (27). Loci with MAF >1% and GF <70% were included in the analysis. The filtered alignment contained 137 814 SNPs. An approximately maximum likelihood phylogenetic tree was estimated with FastTree (28) from the SNP sites in the core alignment (obtained with SNP-sites (29)) using the GTR model of nucleotide substitution and gamma rate heterogeneity among sites.

In contrast to the *S. pneumoniae* alignment, where all sequences were mapped to a reference sequence, this pan-genome-wide alignment was constructed by concatenating individual gene alignments. As a result, we can no longer use a straightforward distance-based cut-off to filter out LD-mediated links. Instead, we simply define two sites within the same gene as an LD-pair and two sites from different genes as a non-LD pair. The main aim for this dataset was to investigate if our method would still be able to extract plausible signals of co-selection under this modified setup.

## RESULTS

### Simulated data

We begin by examining two example cases in more detail. The selected cases were generated under equal-sized populations (50:50), and $\rho /\theta = \ 2$ in the first case and $\rho /\theta = \ 1$ in the second. The number of subsampled sequences in the considered datasets was 400. After reweighting, the effective sample sizes were 37.49 and 26.11, respectively.

**Figure 3.**
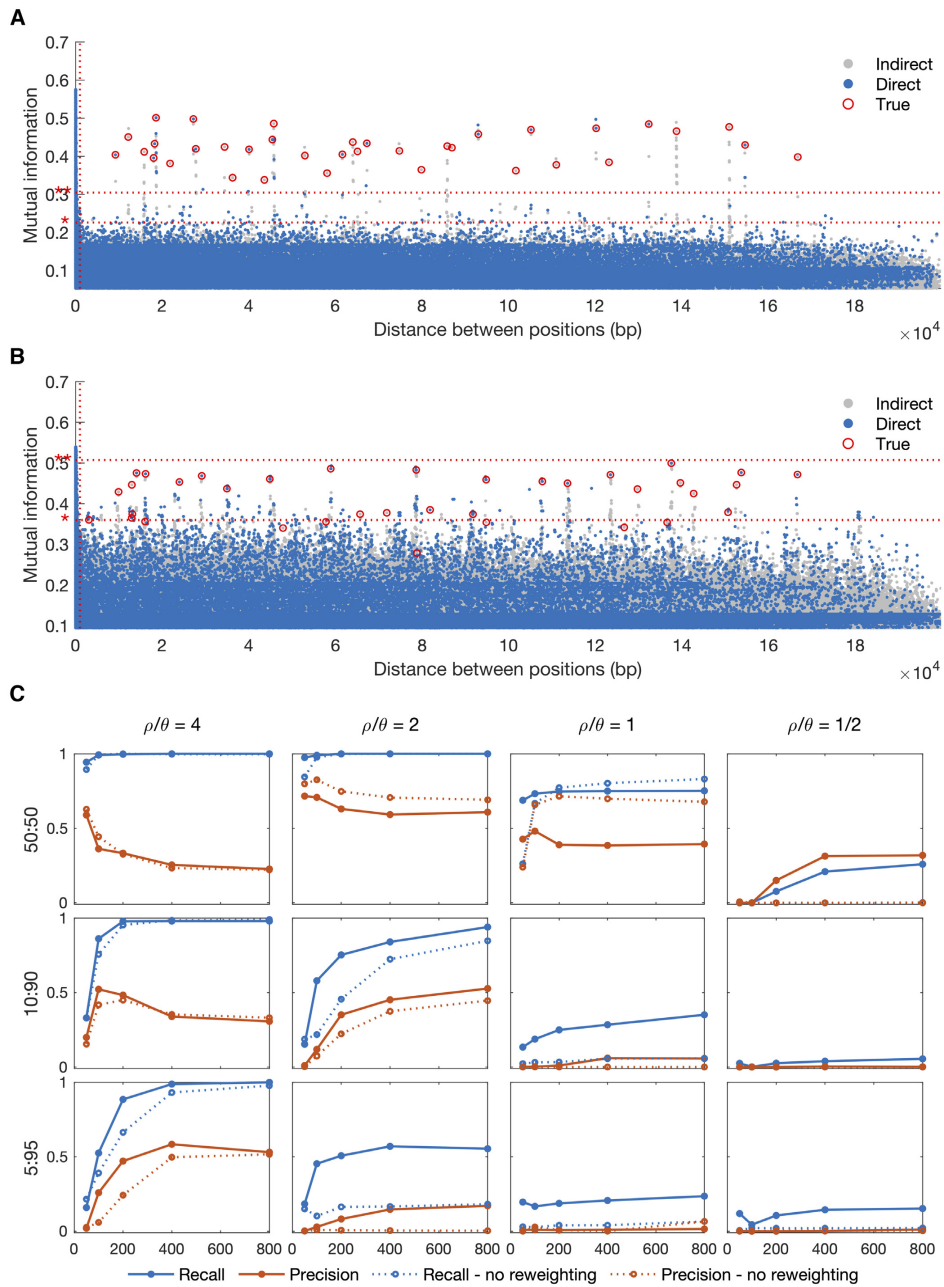
Simulated data: (**A** and **B**) GWES Manhattan plot for two example cases with 400 sequences generated under equal-sized populations and (A) $\rho /\theta = \ 2$ and (B) $\rho /\theta = \ 1$. Direct and indirect links are represented by blue and gray dots, respectively, and true links by red circles. The red horizontal dotted lines show the outlier thresholds; outlier * and extreme outlier **. The red vertical dotted line shows the LD threshold. (**C**) Precision and recall for the outlier links at different sample sizes. Each row of plots represents a population size ratio and each column a $\rho /\theta$ value.

A Manhattan plot illustrating the output of SpydrPick for the first dataset is shown in Figure 3A. The LD threshold, which was set to 1 kbp, is marked with a red vertical line. The lower and upper horizontal red lines in the plot mark the outlier and extreme outlier threshold, respectively. For short-distance SNP pairs there is a peak of high MI values caused by LD. As the distance increases, the background distribution flattens out and remains at a constant level around 0.20. In this particular case, the co-selection signal is clearly separated from the background distributions, and all SNP pairs under co-selective pressure (red circles) exceed even the extreme outlier threshold. In total, 193 direct non-LD links exceeded the outlier threshold, while 87 exceeded the extreme outlier threshold. All nine selected SNPs were found among the extreme outlier links.

The second example case is slightly more challenging, since the recombination rate is lower than in the first case. This is reflected by a lower effective sample size. A Manhattan plot for the second dataset is shown in Figure 3B. While the majority of the true links have similar MI values as in Figure 3A, the background noise is now stronger making it more difficult to separate the signal from the background distribution. In total, 444 direct non-LD links exceeded the outlier threshold, while not a single non-LD link exceeded the extreme outlier threshold. All nine selected SNPs were found among the outlier links.

At last, to perform a more systematic and extensive simulation study, we applied SpydrPick on all 3000 synthetic datasets. To summarize the results, we used modified versions of the concepts of precision and recall. In practice, even after the ARACNE filtering step, each true link is typically accompanied by a collection of links between regions in strong LD with the selected SNPs. To account for these in our accuracy measures, we relax the true positive criterion and consider a position within 500 bp of a selected SNP as a correct hit for that particular SNP. We then define recall as the fraction of unique selected SNPs that are included among the outlier links, that is, a value of 1 means that all selected SNPs were detected. Additionally, precision is defined as the fraction of links among the outlier links that are between selected SNPs, that is, a value of 1 means that all links are between selected SNPs (or between regions around the selected SNPs).

Precision and recall curves for different sample sizes and simulation settings are shown in Figure 3C. While the recall (blue lines) tends to increase with the sample size, the precision (red lines) is a bit more erratic. Still, in all except the most challenging cases, the recall is around 0.20–0.60, which is very reasonable considering the extremely high-dimensional nature of the problem. Moreover, for the simpler cases, where the signal and background were well separated, the extreme outlier links resulted in similar recall but an improved precision (0.60–0.90) indicating that the majority of the underlying signal is captured by the strongest links (Supplementary Figure S1). We also tested the effect of changing the definition of a true hit to specific positions, rather than using regions around positions. While this obviously resulted in a clear drop in precision, the recall largely remained at a similar level, meaning that the specific sites under selection were included among the outlier links in most of the cases (Supplementary Figure S2).

When comparing the results for the different simulator settings, there are two distinct patterns. First, and as already indicated by Figure 3A and B, the success rate increases with a higher recombination rate, which corresponds to a higher value of $\rho /\theta$ in our simulations. Second, a larger skewness between the two populations sizes lowers the success rate. These observations can be expected to apply to GWES in general, rather than being method-specific in terms of what choices are made to measure the dependence and how to account for the population structure. In fact, the importance of a sufficient recombination rate has previously been discussed in similar terms in the context of DCA-based GWES (10,30). Also, the skewness phenomenon is related to previous observations regarding low entropy positions in MI-based co-evolution detection for protein alignments (1,2). As a third and final observation, adjusting for the population structure through sequence reweighting clearly increases the success rate when we have unequal population sizes, which is perhaps the most likely scenario in practice.

Although these experiments provide some insight into the behavior of our method, and the prospects of GWES in general, it is difficult to give some general exact guidelines on the required sample size. The minimum number of samples required will be case-specific, depending on several properties in the data. In these simulations, we got very good results already with a few hundred samples, but this is not necessarily the case in more complicated evolutionary scenarios. In addition to sample size, population diversity is an equally important sample property required for a successful GWES. Consequently, the effective sample size is likely more informative than sheer sample size for assessing the appropriateness of a dataset for GWES (Supplementary Figure S3).

### Streptococcus pneumoniae

**Figure 4.**
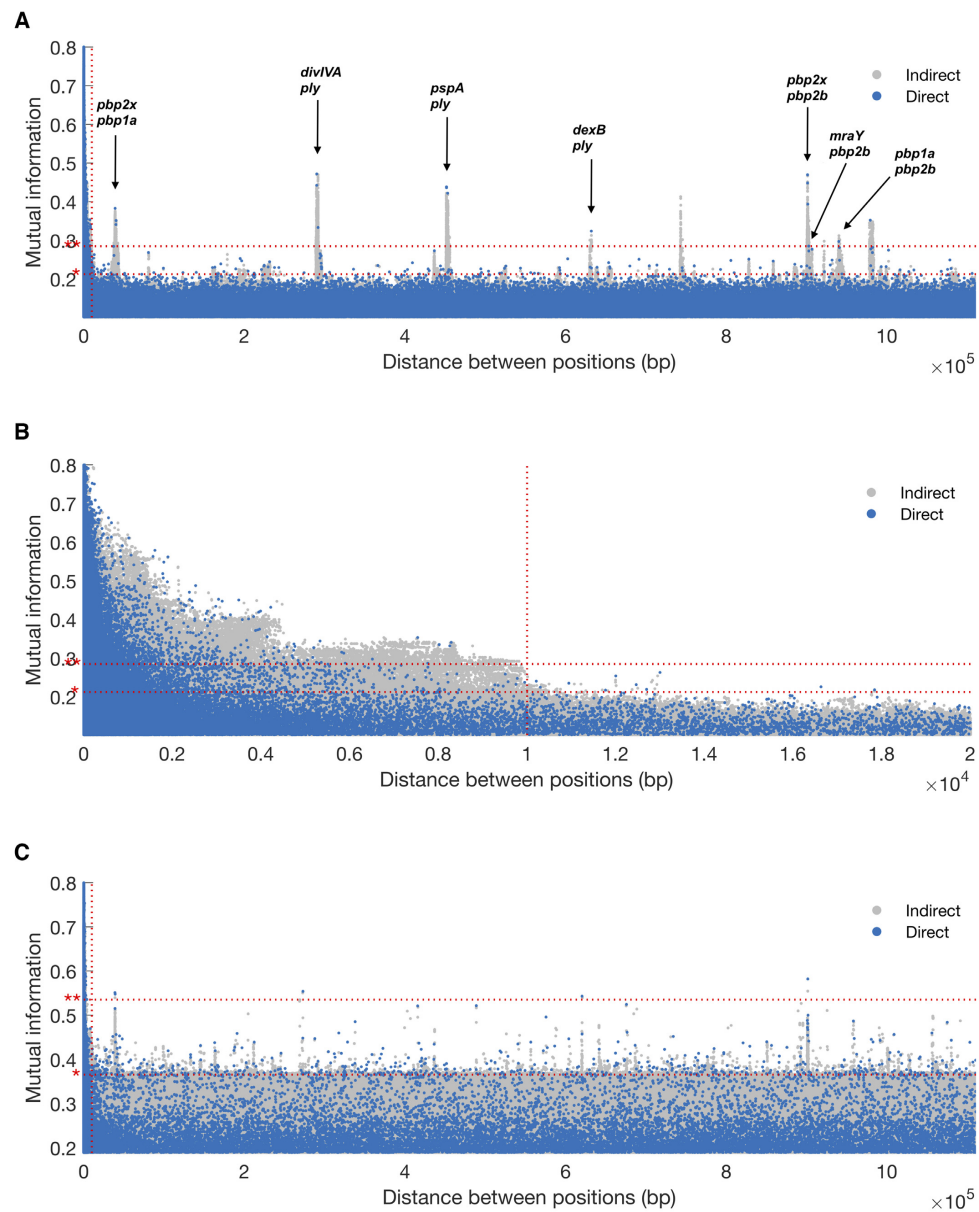
*Streptococcus pneumoniae*: GWES Manhattan plots: (**A**) complete distance range and with annotated peaks, (**B**) distances in the range 0–20 kbp, (**C**) complete distance range but without sequence reweighting. Direct and indirect links are plotted in blue and gray, respectively. The red horizontal dotted lines show the outlier thresholds; outlier * and extreme outlier **. The red vertical dotted line shows the LD threshold.

**Figure 5.**
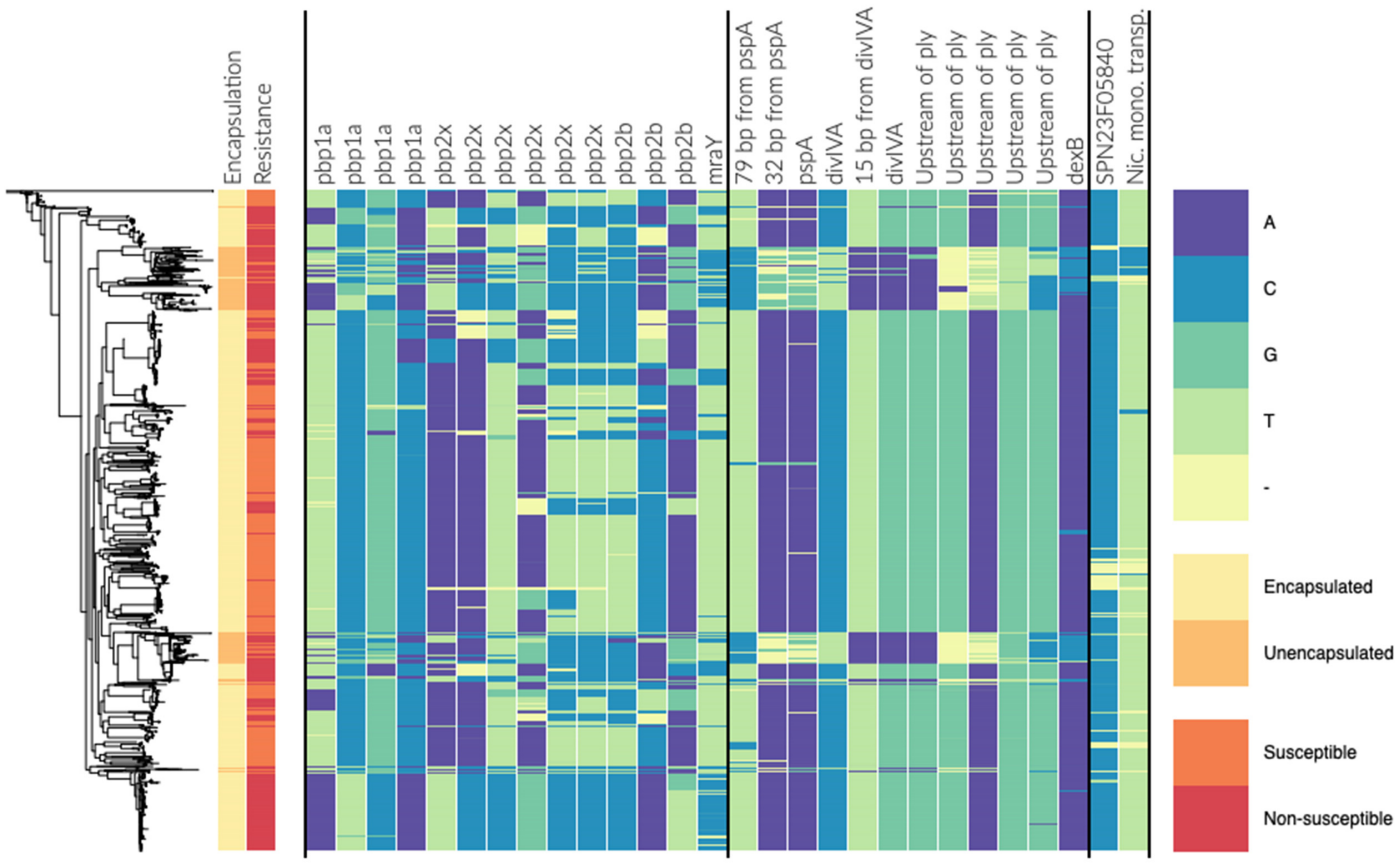
Phenotype information (encapsulation and beta-lactam resistance) and allele distribution at loci involved in the top links for the *Streptococcus pneumoniae* population. The estimated phylogeny is shown on the left. The two first columns are labeled by phenotype information and the remaining columns are labeled by gene name/id. The loci are sorted component-wise such that all columns within two successive vertical lines belong to the same component.

After reweighting with respect to the filtered alignment, the effective sample size was reduced to 130.26. The Manhattan plot of the analysis output is shown in Figure 4A. There is a high LD peak for short-distance pairs flattening out around 10 kbp, which was also used as cut-off value for the LD threshold (see Figure 4B). There are several distinct peaks clearly rising above the background distribution. Each peak is made up of a large collection of potential links. However, the ARACNE step filters out the vast majority as indirect, and only a few representative links (blue points) are singled out for further examination. In total, 163 direct links were flagged as outliers and 16 as extreme outliers. Here, we look closer at the extreme outliers, which are listed in Supplementary Table S2. To facilitate the interpretation of the results, we have annotated the most interesting peaks in the Manhattan plot in Figure 4A using the distance column in Supplementary Table S2. At last, the Phandango plot (31) in Figure 5 shows the allele distributions across the population of the loci involved in the top links alongside phenotypic information about encapsulation and beta-lactam resistance.

The majority of the top-ranking links discovered in the earlier DCA-based GWES (10,11) were between three genes encoding penicillin-binding proteins (PBPs): SPN23F03410 (*pbp1a*), SPN23F16740 (*pbp2b*) and SPN23F03080 (*pbp2x*). These three proteins are involved in cell wall metabolism and are the primary targets of beta-lactam antibiotics. Modification of all three sequences is required for *S. pneumoniae* to exhibit high-level resistance to beta-lactam antibiotics (32–35). Among the top 16 SpyderPick hits, 7 are between PBPs and the corresponding peaks are at distances 0.4 × 10^5^, 9.0 × 10^5^ and 9.4 × 10^5^ bp in the Manhattan plot. In addition to the links between the PBPs, there is also one link from *pbp2b* to SPN23F03090 (*mraY*), which is located directly downstream of *pbp2x*. The *mraY* gene encodes a phospho-N-acetylmuramoyl-pentapeptide-transferase also involved in cell wall biogenesis and, as noted by (10), it has been predicted that mutations in this transferase could be compensating for the costs of evolving beta-lactam resistance (35).

In addition to the PBP-related links, there are four links involving SPN23F19490, which is part of the gene cluster SPN23F19480–19500 located directly upstream of SPN23F19470 (*ply*), encoding the toxin and key virulence factor pneumolysin. The ply-associated gene is coupled with SPN23F16620 (*divIVA*), SPN23F01290 (*pspA*) and SPN23F03150 (*dexB*), corresponding to the peaks at distances 2.9 × 10^5^, 4.3 × 10^5^ and 6.3 × 10^5^ bp in the Manhattan plot. The *divIVA* gene encodes a cell morphogenesis regulator and *pspA* encodes a surface protein associated with virulence. Links between *ply*-associated genes, *divIVA* and *pspA* were discovered as significant by the initial DCA method (10), but not by the more recent DCA method (11). A plausible reason for this is that the more recent DCA method fits a global model over all sites, whereas the initial DCA method uses a subsampling technique that makes it more similar to our local approach. The final link involving *dexB* has not been previously detected by any method. The *dexB* gene is located adjacent to the capsule polysaccharide synthesis locus in most *S. pneumoniae*, suggesting a possible link between the extracellular polysaccharide and the surface-associated PspA, Ply and DivIVA proteins. Further examination revealed the minor alleles at these loci were confined to several phylogenetically distinct clusters of non-typeable (unencapsulated) isolates, which lack a functional capsule polysaccharide synthesis locus (see Figure 5). This suggests non-typeable *S. pneumoniae* are not simply bacteria that have lost their capsule but have also undergone other adaptive changes in specializing to a distinct niche. This may account for the distinct pathogenesis of unencapsulated strains, which do not cause severe invasive disease (36), but are known to cause outbreaks of conjunctivitis (37).

There are two remaining peaks in the Manhattan plot exceeding the extreme outlier threshold. The first peak at distance 7.4 × 10^5^ bp corresponds to an interaction between *pspA* and *divIVA*. This peak is not represented in the top links since it is consistently the weakest link in triplets connecting *ply, pspA* and *divIVA*, and has therefore been labeled as indirect. The second and final peak is an example of a gap-driven signal: the MI of the corresponding link drops from 0.352 to 0.006 when excluding sequences that contain a gap on either site (Supplementary Table S2).

At last, to illustrate the effect of the population structure, the result of running the analysis without sequence reweighting is shown in the Manhattan plot in Figure 4C. When comparing to the original plot in Figure 4A, this further highlights the importance of correcting for the population structure when performing GWES.

### Neisseria meningitidis

**Figure 6.**
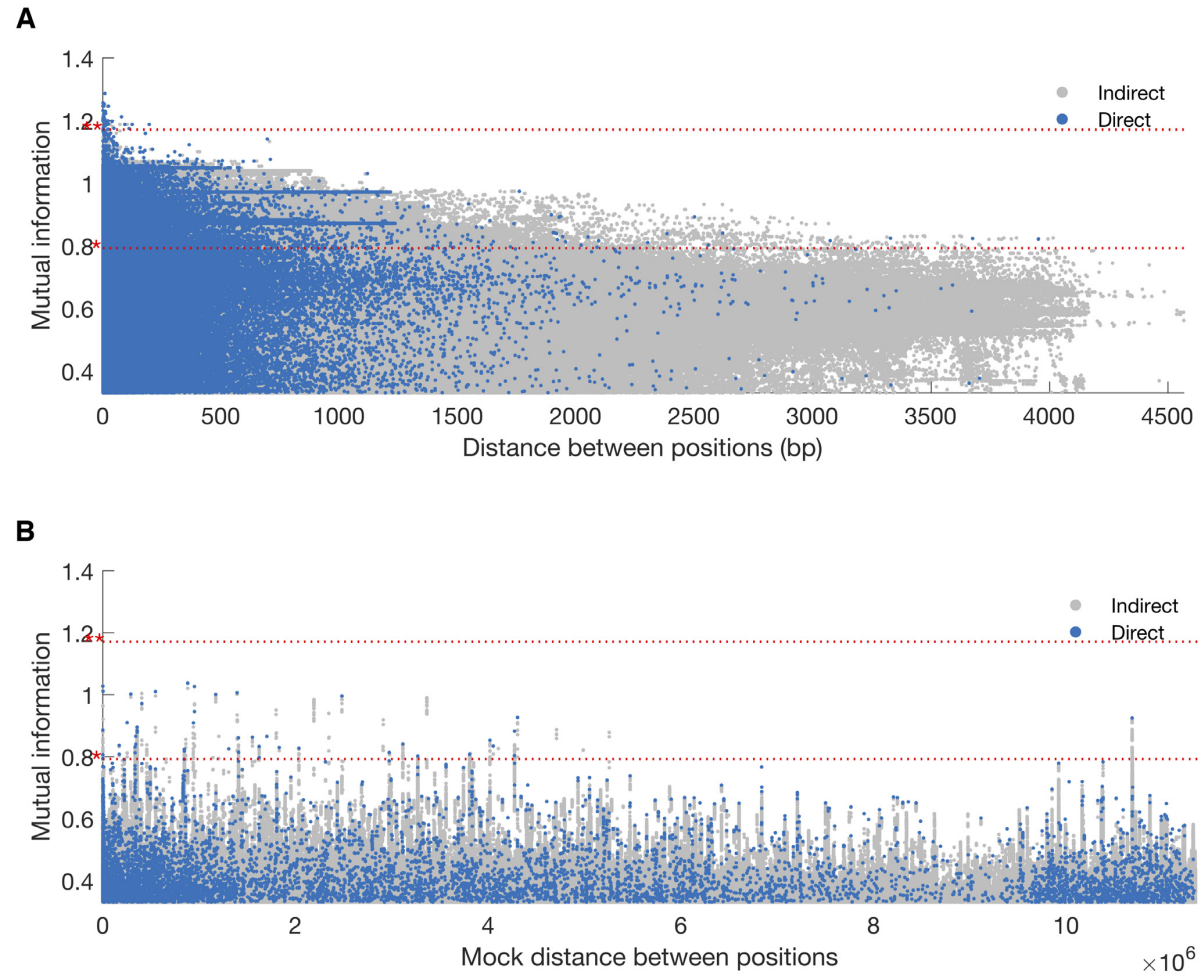
*Neisseria meningitidis*: GWES Manhattan plots: (**A**) intra-gene links, (**B**) inter-gene links. The mock distance in (B) was calculated using the gene order in the actual alignment and is therefore not a true distance. Direct and indirect links are plotted in blue and gray, respectively. The red horizontal dotted lines show the outlier thresholds; outlier * and extreme outlier **.

**Figure 7.**
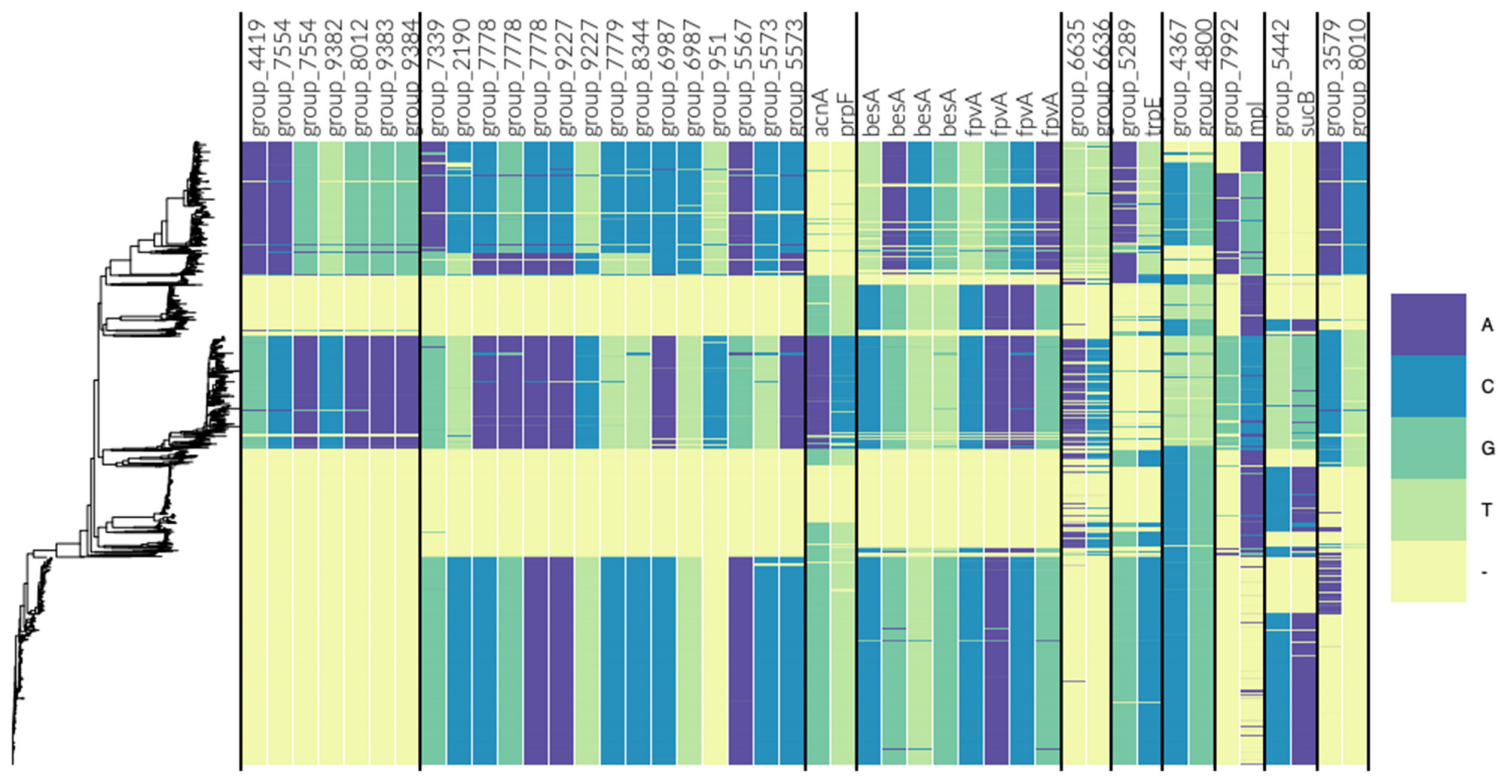
Allele distribution at loci involved in the top links for the *Neisseria meningitidis* population. The estimated phylogeny is shown on the left and each column is labeled by gene name/id. The loci are sorted component-wise such that all columns within two successive vertical lines belong to the same component.

After reweighting with respect to the filtered alignment, the effective sample size was reduced to 515.86. The Manhattan plots for intra- and inter-gene pairs are shown in Figure 6A and B, respectively. Note that the distance between sites in Figure 6B is not a true distance, but a mock distance constructed for illustrative purposes from the ordering of the genes in the alignment. As expected, Figure 6A shows an abundance of high MI values among intra-gene pairs, especially among short-distance pairs. Figure 6B indicates that there is a collection of interaction signals rising above the global background distribution. Still, the overall signal-to-noise (or signal-to-background) ratio appears lower than in the *S. pneumoniae* analysis, which is also reflected by high outlier thresholds. A likely explanation for this is the inclusion of LD-mediated inter-gene links. In total, 48 direct links are flagged as outliers. In the following, we look closer at the 28 top-ranked links, which are listed in Supplementary Table S3. The allele distributions of the loci involved in these links are visualized by the Phandango plot in Figure 7.

The majority of the identified links are between proteins of unknown function, many of which display high similarity to other phage-associated proteins or phage repressors. Previous work has identified a certain bacteriophage as important to virulence in *N. meningitidis* (38,39), but the phage-associated proteins detected in this scan could not be further identified. To better assess the likelihood of LD causing the elevated MI values, we mapped the genes involved in the top links onto the reference genomes MC58 (40) and FAM18 (41), and calculated the inter-gene distances (Supplementary Table S3). This revealed that most of the links were relatively short-distance, making it difficult to rule out the possibility of LD, especially for intra-phage-links. Hence, we looked closer at the five long-distance links for which the involved genes were more than 10 kbp apart in the reference genomes.

Out of the five long-distance links, four links were between the gene *besA*, encoding ferri-bacillibactin esterase and the ferripyoverdine receptor *fpvA*. Both genes are involved in iron uptake during colonisation (42,43). Iron uptake is an important pathway in most bacteria that colonize human hosts, and *Neisseria* is no exception, where iron uptake has been identified as an important determinant of virulence (44,45), and essential for successful colonization (45). The *besA* and *fpvA* genes are located 62 477 bp apart in the MC58 reference genome and 63 514 bp apart in the FAM18 reference genome, and are thus very unlikely to be caused by the background LD.

The final long-distance link is between the anthranilate synthase component I, *trpE*, involved in tryptophan synthesis, and a hypothetical gene, here referred to as *group_5289* (name given by Roary). When searched against the non-redundant protein database with tblastx (46), *group_5289* showed similarities with a betaine transporter. The *trpE* and *group_5289* genes are 361 849 bp apart in the MC58 reference genome and 722 196 bp apart in the FAM18 reference genome. From previous molecular biology work studying these pathways, we can see how these two genes might come to be under selection. Tryptophan synthesis is a crucial part of protein biosynthesis, and its synthesis has been linked to greater virulence in other bacterial species by allowing for immune evasion (47). As for *group_5289*, importing betaine has long been recognized as an important method of surviving in urinary tract infections (48,49), a niche which *N. meningitidis* has long been known to have the ability to infect (50) and appears to be increasing in prevalence (51).

The GWES results have this far been discussed at gene level. Even though SpydrPick outputs links between specific sites, we recommend that the initial examination of the discovered links is kept at gene resolution, since fine-mapping the exact location of SNPs under selection in a GWES is typically very difficult. However, once a link between an interesting gene pair has been identified, one might still want to zoom in and look for further evidence of co-selection at SNP resolution. In particular, when an identified SNP is located in a protein-coding region, one might want to check if the SNP is synonymous or non-synonymous. As an illustrative example, we looked closer at the SNPs involved in the link between *trpE* and *group_5289*. While the SNP in the *group_5289* was found to be non-synonymous, resulting in an arginine to lysine mutation, the SNP in *trpE* was found to be synonymous at the protein-coding level. As synonymous mutations are not typically expected to be under selection, we scanned the surrounding region of the *trpE* site to look for a biologically more likely source of the signal. More specifically, using the SpydrPick output, we extracted all *trpE* sites that were in strong LD (measured by MI) with the original *trpE* site. Using the MI of the original link between *trpE* and *group_5289* as a threshold, we found 14 candidate SNPs located 36–676 bp from the original *trpE* site. Among these, we found one non-synonymous SNP, coding an aspartic acid to alanine mutation. At last, to predict the functional effect of the amino acid substitutions, we used SNAP2 which outputs a value between −100 (completely neutral) and 100 (high functional effect) (52). The predicted effects of the *group_5289* and *trpE* mutations were 45 and 32, respectively, making both likely candidates for mutations under selection.

### Runtime

Running SpydrPick on the *S. pneumoniae* and *N. meningitidis* alignments took 38 and 40 min, respectively, using eight threads on a laptop with Intel Core i7–6820HQ CPU. In comparison, it took over a week for SuperDCA to run direct coupling analysis on the *S. pneumoniae* alignment using a single 20-core dual-socket compute node (11).

## DISCUSSION

The rapidly increasing availability of population-wide genome sequence data has boosted the potential for data-driven exploration of genetic variation associated with bacterial evolution. As a result, high-dimensional exploratory data analysis methods have become valuable tools to help generate detailed hypotheses and identify important targets for subsequent experimental work. For eukaryotes, genome-wide association studies (GWAS) have been the primary tool for this purpose for more than a decade, and more recent works have demonstrated the applicability and potential of GWAS also for bacteria (35,53,54). In addition to GWAS, the phenotype-free approach of GWES has recently emerged, and successfully been used to uncover mechanisms behind complex bacterial traits associated with survival, proliferation and virulence (9–11).

The main advantage of GWES lies in its unsupervised approach. It does not require the definition and measurement of a phenotype, yet it can reveal co-evolutionary patterns behind many different traits shaped by selection. Bacterial genomes of a single species are likely sampled from diverse micro-niches, which create unique selective pressures that vary over space and time. These can include immune pressures, nutrient availability, antibiotic use or interactions within ecological communities. Links identified by GWES may represent multilocus adaptation to these micro-niches, which will create combinations of mutations that are maintained by selection. This adaptive process may be facilitated by epistatic interactions between loci but may also be driven by independent selection on sets of mutations that are additively beneficial in a particular niche. Co-evolutionary signals may also be maintained in a population if negative frequency dependent selection (NFDS) acts on the same traits. In fact, it has recently been suggested that NFDS acts to prevent antibiotic resistance genes sweeping to fixation in *S. pneumoniae* (55).

In this work, we introduced the model-free GWES method SpydrPick, which is parallelizable and scalable to pan-genome-wide alignments of many bacteria. To illustrate the output of a GWES, we introduced a modified version of the Manhattan plot, which has served as the main illustrative tool for exploring the output of GWAS. While simulations validated our method in a controlled setting, the analysis of an *S. pneumoniae* alignment showed that SpydrPick was able to accurately pick out previously discovered and validated signals of co-selection, as well as a novel link with a plausible biological explanation. In addition, a pan-genome-wide analysis of a Roary generated alignment of *N. meningitidis* isolates illustrated the potential of our method for an even more challenging dataset, by identifying several interesting signals likely to originate from genes under selection.

Similar to previous GWES methods, SpydrPick operates on SNP resolution trying to fine-map the co-selection signal to individual sites using only the co-variation pattern observed in the data. For any method, this task is very challenging and limited by several factors, such as population structure, extent of LD and amount of available data. As illustrated by the identified *trpE* site in *N. meningitidis*, it is likely to be informative to check the surrounding region of the statistically linked sites to find the biologically most plausible source of the signal.

Compared to model-based DCA methods that aim to fit a joint model over all SNPs, SpydrPick is conceptually very different in that each pairwise interaction between two sites is evaluated independently of all other sites. This is similar in spirit to the approach by Cui *et al.* (9), who used Fisher's exact test to scan for epistatic interactions among bi-allelic SNPs in a sample of *Vibrio parahaemolyticus* isolates. In contrast to our method, however, Cui *et al.* did not attempt to disentangle the direct interaction from the indirect interactions. In a recent hybrid approach, Gao *et al.* proposed filtering the data based on pairwise correlations and then fitting a joint model over the remaining sites in (56). The obvious advantage of a strict pairwise method, such as SpydrPick, is that its computational simplicity allows for scaling up to datasets beyond what is currently achievable by current DCA-based methods. In addition, and more importantly, recent numerical experiments on synthetic network models suggest that pairwise methods may be more accurate than the current state-of-the-art DCA-based methods in the high-dimensional setting (12).

To distinguish between LD and non-LD links, we used a distance-based threshold. As the background distribution will depend on multiple factors, such as type of organism, mode of recombination, population structure of the sample etc., it might be necessary to adjust the threshold value accordingly. This may involve running the analysis twice, where the output of the initial run is solely used to re-adjust the LD threshold parameter according to the drop in LD observed in the Manhattan plot (see Figure 4B). A topic for future research will be to look into alternative and more sophisticated means for distinguishing between LD and non-LD links. This will be particularly important for alignments where a distance-based threshold cannot be used easily, for example in the analysis of the pan-genome. However, it might also open up opportunities for identifying signals of co-selection between closely located SNPs.

Another important topic for future research is to compare different techniques for adjusting for the population structure, as the optimal technique will most likely depend on certain properties in the data, such as the level of clonality among the isolates. In the work by Cui *et al.* (9), a subsample of 51 unrelated isolates was selected for the co-variation analysis, meaning that each collection of closely related isolates in the original dataset was represented by a single isolate. In contrast, the conceptual idea behind the reweighting technique used here can essentially be thought of as taking the average over the same collections of isolates. In the study by Dutheil (13), incorporating evolution-awareness into the correlation statistic was shown to be more effective than data filtering alone in terms of correcting for shared ancestry when inferring co-evolving pairs from bacterial RNA data. Consequently, data filtering was recommended to be used primarily for computational reasons, and preferably in combinations with evolution-aware methods. An important aspect of the built-in population structure correction of the reweighted MI estimator is that it does not require an explicit phylogenetic tree, or any other form of model. Instead, it uses the phylogenetic signal present in the data, enabling scalability to the genome-wide setting and avoiding potential issues related to model misspecification.

GWES is a relatively new data-driven and phenotype-free approach for detecting co-evolutionary patterns shaped by selection, and it is currently gaining traction in bacterial genomics due its wide applicability. Although GWES is a stand-alone analysis, there is also a lot of potential in combining it with other well-established methods. For example, if one has access to relevant phenotypic data, the output of a GWES can be used to effectively reduce the number of tests in a follow-up epistatic GWAS (57). Given its accuracy and computational scalability, SpydrPick pushes the boundaries of existing GWES methods and promises to uncover a wealth of previously-undiscovered evolutionary signals in bacterial genomic data.

## DATA AVAILABILITY

The SpydrPick software is available from GitHub: https://github.com/santeripuranen/SpydrPick, as well as the Anaconda repository: https://anaconda.org/bioconda/spydrpick. An MSA of the *S. pneumoniae* strains is available from the Dryad Digital Repository: https://datadryad.org/resource/doi:10.5061/dryad.gd14g. The *N. meningitidis* strains are available from the European Nucleotide Archive and their accession numbers are given in Supplementary Table S1.

## Supplementary Material

gkz656_Supplemental_Files
