# Supplementary material for: Genome-wide epistasis and co-selection study using mutual information
Source: Nucleic Acids Res. 2019 Jul 30;47(18):e112. doi: 10.1093/nar/gkz656 (PMC6765119; doi:10.1093/nar/gkz656)
Supplement: gkz656_Supplemental_Files [file gkz656_supplemental_files.zip › Figures S1-S3.pdf]

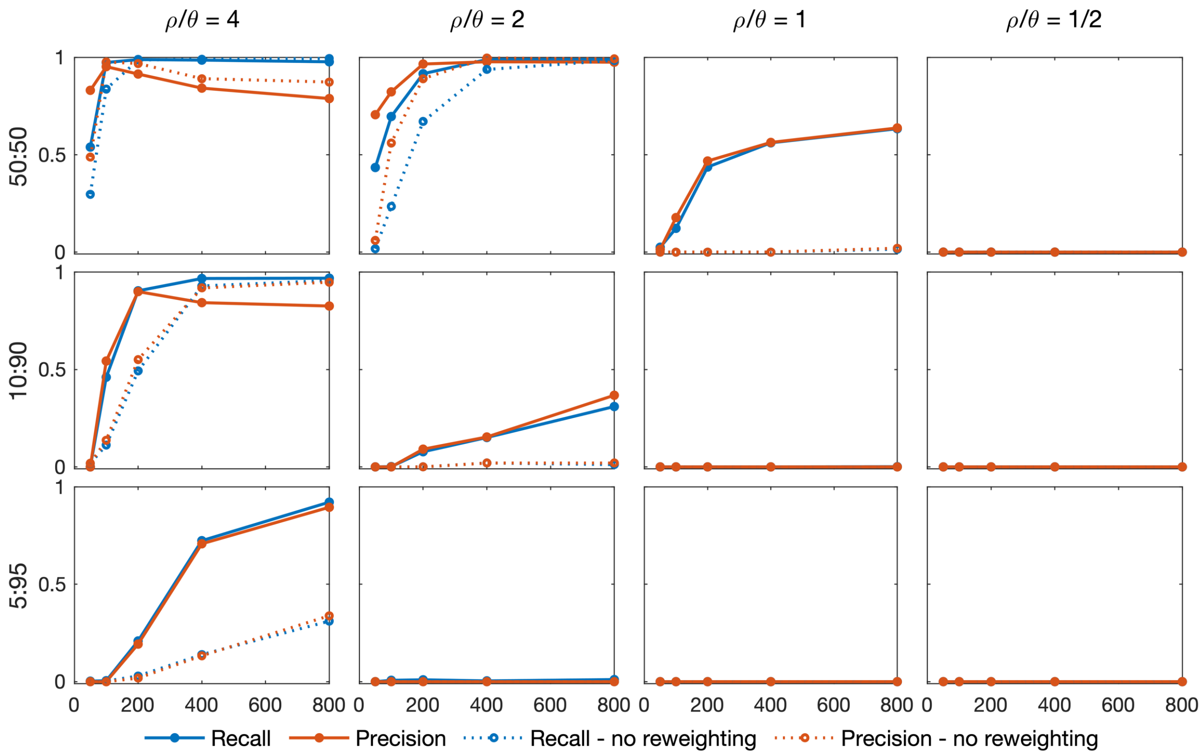

Figure S1. Simulated data: Precision and recall for the extreme outlier links at different sample sizes. Each row of plots represents a population size ratio and each column a ρ/θ value.

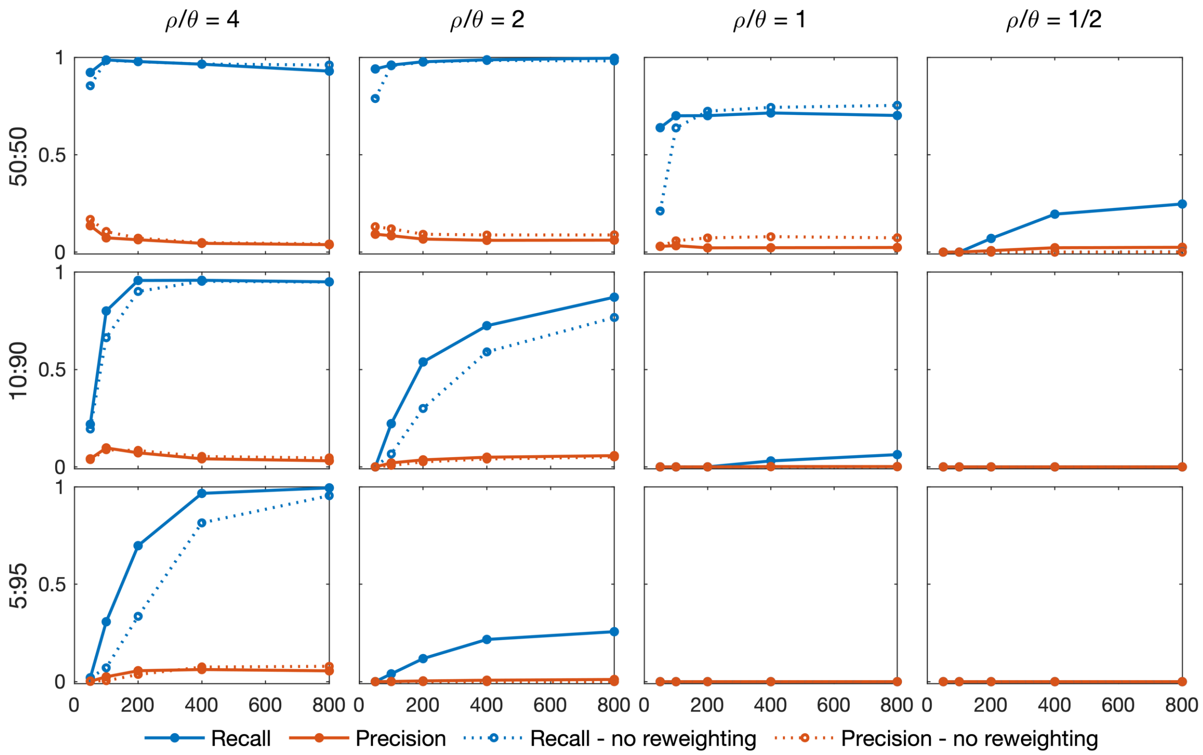

Figure S2. Simulated data: Precision and recall for the outlier links at different sample sizes when only the specific positions under selection are considered true positives. Each row of plots represents a population size ratio and each column a ρ/θ value.

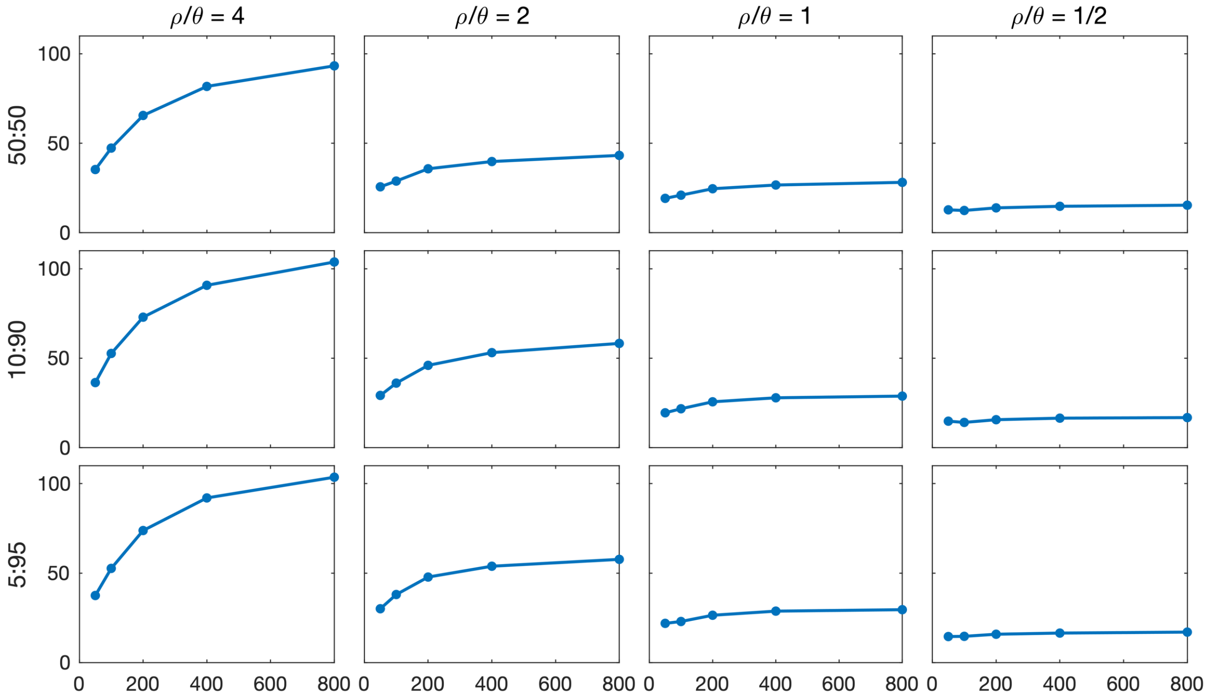

Figure S3. Simulated data: effective sample size (y-axis) vs sample size (x-axis). Each row of plots represents a population size ratio and each column a ρ/θ value.
